# Nutritional Issues in the Critically Ill Patients; Challenges and Conundrums

**Published:** 2017

**Authors:** Majid Mokhtari

The article “An Iranian Consensus Document for Nutrition in Critically Ill Patients, Recommendations and Initial Steps toward Regional Guideline Development” which was published in current issue of Tanaffos has raised many important concerns regarding different aspects of nutritional challenges in the critically ill ICU patients.

The authors have tried to highlight the importance of the early, adequate and proper nutrition in the ICU patients. This group of patients with their increasing diversities, are at ever increasing risk of myriads of complications, which stem from ICU nutritional errors. The following comments and suggestions, regarding this document, may prove useful for the future endeavours of this nature.

The manner this guideline and recommendations is structured, creates some confusion, where the general and basic knowledge of energy/metabolic requirements and nutritional assessment elaborated in a manner not exactly adherent to the standard guideline formats. Many aspects of these recommendations are not suitable to the critically ill ICU patients and are merely reiteration of the basic knowledge used for the nutritional assessment of all patient’s groups.

Some recommendations included in this document, particularly for nutritional assessment, are not practiced commonly in the critical care units of the developed countries, let alone globally, particularly in the resource poor nations. For example, the application of indirect calorimetry and other tools mentioned, which require appropriate equipment and personnel, in many ICU’s where there is not even patient’s weighing tools available, seems quite irrelevant.

Simpler, more readily available and validated nutritional assessment and feeding tools such as NUTRIC score, CAN WE FEED protocol or PEPuP protocol could have been highlighted as more clinically applicable for daily use in the ICU.

Other practical aspects of nutrition in the critically ill patients such as enteral feed intolerance, ongoing issues with the gastric residual volume, continuous vs. interrupted feeding, compensatory feeding, volume based feeding, nurse driven protocols, role of supplementary parenteral feeding, addressing ways to prevent unnecessary feeding interruptions and iatrogenic starvation could have been addressed in this document.

As an attempt to heighten the awareness of the critical care physicians in the country, the authors are encouraged to consider this document as a base ground to build on, for the next versions of their local guideline.

My gratitude to the authors for their effort,
